# Distinctive Temporal Trajectories of Alzheimer’s Disease Biomarkers According to Sex and *APOE* Genotype: Importance of Striatal Amyloid

**DOI:** 10.3389/fnagi.2022.829202

**Published:** 2022-02-07

**Authors:** Jun Pyo Kim, Min Young Chun, Soo-Jong Kim, Hyemin Jang, Hee Jin Kim, Jee Hyang Jeong, Duk L. Na, Sang Won Seo

**Affiliations:** ^1^Department of Neurology, Samsung Medical Center, Sungkyunkwan University School of Medicine, Seoul, South Korea; ^2^Center for Neuroimaging, Radiology and Imaging Sciences, Indiana University School of Medicine, Indianapolis, IN, United States; ^3^Department of Health Sciences and Technology, SAIHST, Sungkyunkwan University, Seoul, South Korea; ^4^Department of Intelligent Precision Healthcare Convergence, Sungkyunkwan University, Suwon, South Korea; ^5^Alzheimer’s Disease Convergence Research Center, Samsung Medical Center, Seoul, South Korea; ^6^Department of Digital Health, SAIHST, Sungkyunkwan University, Seoul, South Korea; ^7^Departments of Neurology, Ewha Womans University Seoul Hospital, Ewha Womans University College of Medicine, Seoul, South Korea; ^8^Stem Cell & Regenerative Medicine Institute, Samsung Medical Center, Seoul, South Korea

**Keywords:** Alzheimer’s disease, trajectory curve, sex, apolipoprotein E, positron emission tomography

## Abstract

**Purpose:**

Previously, sex and apolipoprotein E (*APOE*) genotype had distinct effects on the cognitive trajectory across the Alzheimer’s disease (AD) continuum. We therefore aimed to investigate whether these trajectory curves including β-amyloid (Aβ) accumulation in the cortex and striatum, and tau accumulation would differ according to sex and *APOE* genotype.

**Methods:**

We obtained 534 subjects for ^18^F-florbetapir (AV45) PET analysis and 163 subjects for ^18^F-flortaucipir (AV1451) PET analysis from the Alzheimer’s Disease Neuroimaging Initiative database. For cortical Aβ, striatal Aβ, and tau SUVR, we fitted penalized splines to model the slopes of SUVR value as a non-linear function of baseline SUVR value. By integrating the fitted splines, we obtained the predicted SUVR curves as a function of time.

**Results:**

The time from initial SUVR to the cutoff values were 14.9 years for cortical Aβ, 18.2 years for striatal Aβ, and 22.7 years for tau. Although there was no difference in cortical Aβ accumulation rate between women and men, striatal Aβ accumulation was found to be faster in women than in men, and this temporal difference according to sex was more pronounced in tau accumulation. However, *APOE* ε4 carriers showed faster progression than non-carriers regardless of kinds of AD biomarkers’ trajectories.

**Conclusion:**

Our temporal trajectory models illustrate that there is a distinct progression pattern of AD biomarkers depending on sex and *APOE* genotype. In this regard, our models will be able to contribute to designing personalized treatment and prevention strategies for AD in clinical practice.

## Introduction

Alzheimer’s disease (AD) is the most common cause of dementia, characterized by the accumulation of amyloid-β (Aβ) plaques and neurofibrillary tangles formed by high levels of phosphorylated tau (Sperling et al., 2011). According to the amyloid cascade hypothesis, the deposition of Aβ plaques leads to the development of neurofibrillary tangles, cortical atrophy, and cognitive impairment (Price and Morris, 1999; Jack et al., 2018). Clarifying temporal trajectories of AD biomarkers would be crucial to better understand the disease. In this regard, accumulation of longitudinal positron emission tomography (PET) scans or cerebrospinal fluid (CSF) data has made it possible to model temporal changes in Aβ and tau biomarkers (Mattsson et al., 2012; Hanseeuw et al., 2019). It is a challenging task to clearly demonstrate the general pattern of pathological progression over decades, as the durations of follow-ups of individual subjects are relatively limited. However, modeling of AD biomarker trajectories remains an important goal, as future treatments targeting Aβ and tau are expected to be developed.

One way to overcome the limited individual follow-up period is to group subjects who share similar properties, such as initial clinical diagnosis (Hadjichrysanthou et al., 2020; Cho et al., 2021) or the shape of their longitudinal trajectory (Koscik et al., 2020). Alternatively, an integration-based method (Hahn, 1991) can be used to obtain the calculated time curves of the biomarkers (Jack et al., 2013; Villemagne et al., 2013; Baek et al., 2020a). This method does not require subjects to be grouped, which makes the process of analysis more straightforward. Using this method, Jack et al. proposed a temporal trajectory model of Aβ accumulation using serial amyloid PET scans, showing that Aβ accumulation exhibits a sigmoidal shape across time (Jack et al., 2013). In fact, a recent study showed that this model of Aβ and tau accumulation matches the hypothetical model that shows the orderly appearance of AD biomarkers (Baek et al., 2020a).

According to recent studies using amyloid PET data, Aβ accumulation occurs first in the neocortex and then in the striatum, suggesting a downward spreading pattern consistent with Thal Aβ pathology staging (Thal et al., 2002; Cho et al., 2018; Hanseeuw et al., 2018). Furthermore, the striatal Aβ is associated with worse AD biomarkers and cognitive function (Cho et al., 2018; Hanseeuw et al., 2018). Therefore, we propose that it would be worthwhile to investigate Aβ accumulation in the striatum as well. It is especially important to investigate how the striatal Aβ trajectory is positioned relative to the cortical Aβ and tau trajectories, as it may provide us with more insight regarding AD pathobiology.

In addition, the trajectory of pathological tau accumulation is studied with tau PET data. The transition from normal aging to preclinical AD can be characterized by tau tangles that spread from the medial temporal lobe to limbic areas (Braak stages III and IV) (Petersen et al., 2006). Especially, increased tau uptakes in the Braak stages III and IV regions may reflect pathological tau accumulation. Tau accumulation in Braak stages I and II may occur before the evidence of Aβ accumulation. Also, tau accumulation in Braak stages I and II may be found even in people at a young age or in normal cognitive function or primary age-related tauopathy (Braak and Del Tredici, 2015; Zhu et al., 2019). In fact, many studies calculated the cut-off values of tau positivity using the tau uptakes in the Braak stages III and IV (Maass et al., 2017).

Several factors may affect the cognitive trajectory. Specifically, a previous study from our group showed that sex and apolipoprotein E (*APOE*) genotype had distinct effects on the cognitive trajectory across the AD continuum (Cho et al., 2021). However, in previous studies, *APOE* ε4 carriers with normal cognition showed more Aβ uptake in the cerebral cortex than that by non-carriers (Oveisgharan et al., 2018; Buckley et al., 2019), but these studies did not show women with normal cognition to have more Aβ uptake in the cerebral cortex than men. Therefore, this raised the question as to whether AD biomarkers begin to show different trajectories, including uptakes of cortical Aβ, striatal Aβ, and tau, according to sex or *APOE* ε4 genotype.

In the present study, we aimed to model the temporal trajectories of pathological tau accumulation as well as Aβ accumulation in the cortex and striatum. We also investigated whether these trajectory curves would differ according to sex and *APOE* genotype. We hypothesized that women would show faster progression of AD biomarker accumulation than men in case of striatal Aβ and tau, but not in case of cortical Aβ. We also hypothesized that *APOE*ε4 carriers would show faster progression in accumulation than non-carriers, regardless of the AD biomarker type.

## Materials and Methods

### Data Acquisition

Data used in the present study were obtained from the Alzheimer’s Disease Neuroimaging Initiative (ADNI) database led by Principal Investigator Michael W. Weiner, MD. ADNI is the outcome of a public-private partnership started in 2003. Its main goal is to test whether clinical and cognitive assessment, PET, CSF, serial magnetic resonance imaging (MRI), and other biological markers can be combined to evaluate the progression of mild cognitive impairment (MCI) and early AD dementia (ADD).

### Study Participants

The present study included subjects from the ADNI dataset. Detailed inclusion and exclusion criteria for the ADNI data are provided on the ADNI website^1^. Cognitive function of the participants was evaluated using the Alzheimer’s Disease Assessment Scale Cognitive subscale 13. For trajectory analysis of cortical and subcortical Aβ, subjects who underwent ^18^F-florbetapir (AV45) PET tests were selected. Similarly, subjects who underwent ^18^F-flortaucipir (AV1451) PET were included in the tau trajectory analysis. Information on *APOE* genotype was also investigated.

**FIGURE 1:**
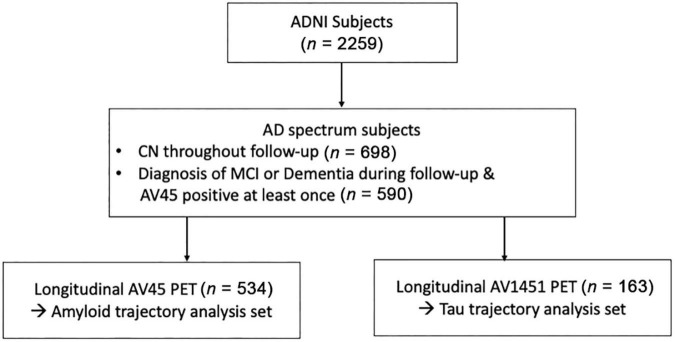
Flowchart of enrolled participants within the study. Abbreviations: ADNI, Alzheimer’s disease neuroimaging initiatives; AD, Alzheimer’s disease; MCI, mild cognitive impairments; CN, cognitively normal; PET, positron emission tomography.

Among the subjects who underwent corresponding PET for each biomarker, we included subjects within the AD continuum from “amyloid-negative, cognitively normal subjects” to “amyloid-positive, demented subjects.” To this end, we included subjects who showed Aβ positivity at least once, regardless of cognitive function. In addition, subjects who remained cognitively normal throughout the follow-up period were included, regardless of Aβ positivity. Subjects with cognitive impairment who did not show Aβ positivity within the follow-up period were excluded (Figure 1).

All participants provided written informed consent and underwent protocols approved by the institutional review board of each participating site. The data use and publication by authors were approved by the ADNI Data Sharing and Publications Committee.

### Image Data Acquisition and ^18^F-Florbetapir Positron Emission Tomography Preprocessing

The ADNI PET acquisition protocols are described on www.adni-info.org. In terms of ^18^F-flortaucipir PET, we obtained standardized uptake value ratios (SUVRs) for Braak stages III and IV from the UCBERKELEYAV1451_PVC table as part of the ADNIMERGE R package. For the analysis of amyloid PET scans, we downloaded the ^18^F-florbetapir PET and corresponding MRI image files from the LONI website and processed them. ^18^F-florbetapir images consisted of 4 × 5 min frames acquired 50–70 min post-injection; these were realigned, averaged, resliced to a common voxel size (1.5 mm × 1.5 mm × 1.5 mm), and smoothed to a common resolution of 8 mm^3^. All T1 images were preprocessed using CIVET pipeline. Segmentation was performed by ANIMAL segmentation in the implemented pipeline (Collins et al., 1994; Collins et al., 1995). The region-based voxel-wise correction method was used for partial volume correction (PVC) and performed using the PETPVC toolbox into total ^18^F-florbetapir PET images (Thomas et al., 2011; Thomas et al., 2016). The ^18^F-florbetapir PET images were co-registered onto corresponding T1 images, and SUVRs were calculated for partial volume-corrected PET images. The whole cerebellum was used as the reference region.

### Trajectory Analysis

**FIGURE 2:**
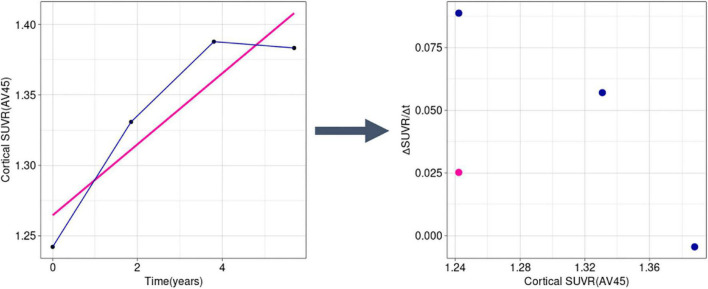
Example transformation of longitudinal SUVR data into ΔSUVR/Δt values in a sample subject (RID: 545). Instead of using a single mean slope value (the pink line and dot), we used all observations separately (dark blue lines and dots), accounting for the change of rate over time. SUVR, standardized uptake value ratio.

We used an in-house program implemented in R for trajectory analysis using a modified method of the previously used method to determine the temporal trajectory of AD-related biomarkers (Jack et al., 2013; Villemagne et al., 2013; Baek et al., 2020a). We calculated the rates of change for each biomarker, using longitudinally observed values. Instead of obtaining a single rate value (ΔSUVR/Δt) per subject, we calculated the slope for every interval, thus obtaining at *n*-1 rate values for a subject with *n* observations (Figure 2). Using this method, we were able to capture the change in rate within a subject throughout the follow-up period.

Thereafter, for each biomarker, we fitted a penalized spline to model the slope of the SUVR as a non-linear function of the baseline SUVR. Penalized splines can be an efficient tool to describe complex non-linear relationships because researchers do not have to determine the amount and placement of the knots. Instead, penalized splines use a large number of knots and allow the fit to be controlled by a penalty (Perperoglou et al., 2019). We used the *gam* function in the *mgcv* R package to fit the penalized splines with the extended Fellner Schall optimization method (Wood and Fasiolo, 2017), and other parameters were set to default settings. The default basis for smoothing by the *gam* function is the thin plate regression spline, which uses knots as many as the number of unique values of the data up to 2,000, placed on every unique value (Wood, 2003).

Using the fitted splines, we obtained predicted SUVR curves as a function of time by using the *ode* function in the *deSolve* R package to solve the first-order differential equation. For each variable, the initial SUVR for time = 0 was anchored to the mean SUVR of amyloid-negative, cognitively normal subjects who underwent the test. The number of subjects and the mean (standard deviation) values of the normative groups are shown in Table 1. The FreeSurfer-based SUVR cortical cutoff of 1.11, calculated based on the same ADNI data (Landau and Jagust, 2015), was found to be 1.043 for the CIVET-based cortical SUVR by regression analysis. The cortical Aβ cutoff of 1.043 corresponded to a *z*-score of −2.0, so the cutoff values of striatal Aβ and tau were set to z-score -2.0. We indicated the abnormal cutoff (*z*-score −2.0) values, the mean SUVRs of MCI subjects, and the mean SUVRs of ADD subjects for each trajectory model.

**TABLE 1:** Normative values used for *z*-transformation.

	***N***	**Mean**	**SD**
Amyloid (cortex)	204	0.863	0.091
Amyloid (striatum)	204	1.58	0.159
Tau (Braak 3,4)	177	1.69	0.144

*N, number of amyloid-negative, cognitively normal subjects used to derive normative values; SD, standard deviation.*

## Results

### Study Participants

We included a different number of subjects for each biomarker’s final analysis because we included all AD continuum subjects with longitudinal data for the given biomarker. For example, while a total of 534 subjects were included in the trajectory analysis of amyloid PET, 163 subjects were available for tau PET trajectory analysis. Demographic data are summarized in Table 2.

**TABLE 2:** Characteristics of analyzed subjects by test modality and biomarker.

	**^18^F-florbetapir (AV45) PET**	**^18^F-flortaucipir (AV1451) PET**
No.	534	163
**Diagnosis (%)**		
CN	297 (55.6)	107 (65.6)
–Dementia	42 (7.9)	18 (11.0)
–MCI	195 (36.5)	38 (23.3)
Age, mean (SD), y	74.0 (6.9)	75.2 (7.5)
Men (%)	282 (52.8)	81 (49.7)
Education, mean (SD), y	16.22 (2.72)	16.39 (2.48)
*APOE* ε4 carrier (%)	253 (47.4)	82 (50.6)

*PET, positron emission tomography; N, number; CN, cognitively normal; MCI, mild cognitive impairment; SD, standard deviation.*

### Rate of Change of Biomarkers According to Baseline SUVRs

**FIGURE 3:**
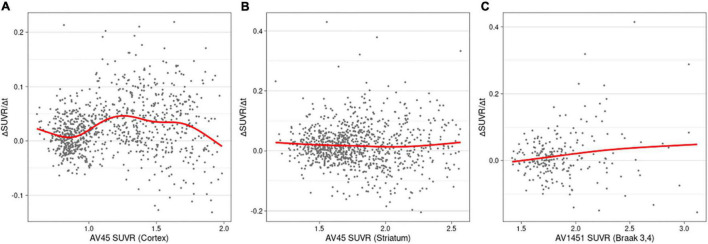
Rates of change (worsening) as a function of SUVRs in cortical amyloid **(A)**, striatal amyloid **(B)**, and pathological tau **(C)**. AV45, ^18^F-florbetapir; SUVR, standardized uptake value ratio; AV1451, ^18^F-flortaucipir.

The rates of changes in the SUVRs for the biomarkers are shown in Figure 3. The cortical and striatal Aβ accumulation rates showed different patterns. While the accumulation rate of cortical Aβ increased until the SUVR reached 1.26 and decreased subsequently (Figure 3A), ΔSUVR/Δt for the striatal Aβ remained constant throughout the entire range of SUVRs (Figure 3B). The worsening rate of tau PET also remained substantially constant (Figure 3C), although tau accumulation showed a slightly increasing pattern.

### Temporal Trajectories of Alzheimer’s Disease Related Markers

**FIGURE 4:**
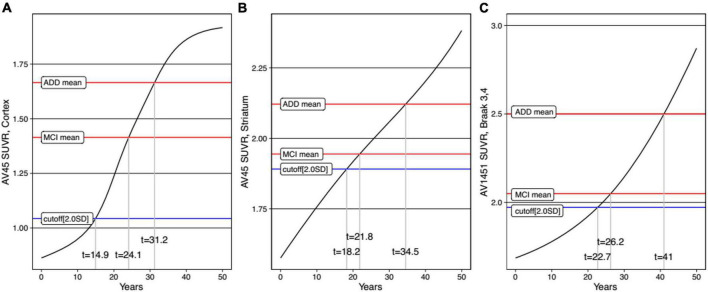
Trajectories of AD biomarkers as a function of time in cortical amyloid **(A)**, striatal amyloid **(B)**, and pathological tau **(C)**. AV45, ^18^F-florbetapir; SUVR, standardized uptake value ratio; AV1451, ^18^F-flortaucipir.

For each variable, we obtained the curve of the expected SUVRs as a function of time (Figure 4). The time from initial SUVR to the cutoff values (*z*-score −2.0) was the longest for tau PET (22.7 years) and shortest for cortical Aβ (14.9 years). For the cortical Aβ, striatal Aβ, and tau trajectories, after reaching the cutoff, the mean SUVR of MCI subjects and the mean SUVR of ADD subjects appeared.

**FIGURE 5:**
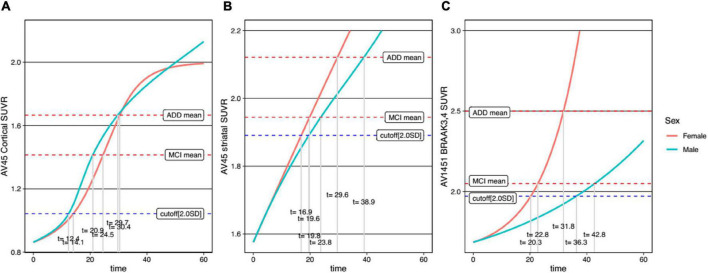
Temporal trajectories of AD biomarkers stratified by sex in cortical amyloid **(A)**, striatal amyloid **(B)**, and pathological tau **(C)**. AV45, ^18^F-florbetapir; SUVR, standardized uptake value ratio; AV1451, ^18^F-flortaucipir; MCI, mild cognitive impairment; ADD, Alzheimer’s disease dementia.

**FIGURE 6:**
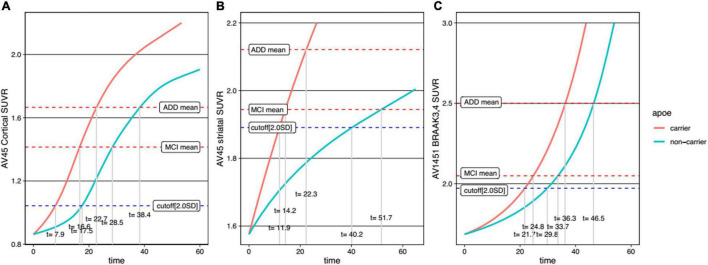
Temporal trajectories of AD biomarkers stratified by APOE genotype in cortical amyloid **(A)**, striatal amyloid **(B)**, and pathological tau **(C)**. AV45, ^18^F-florbetapir; SUVR, standardized uptake value ratio; AV1451, ^18^F-flortaucipir; MCI, mild cognitive impairment; ADD, Alzheimer’s disease dementia.

For comparison between men and women, trajectory curves were generated separately, according to sex (Figure 5). There was no significant difference in the cortical Aβ accumulation rate between women and men (Figure 5A). However, for striatal Aβ accumulation, women reached the cutoff values 2.7 years earlier than men (Figure 5B). The temporal difference according to sex was more pronounced for tau accumulation, as women reached the cutoff 16.0 years earlier than men (Figure 5C). In terms of *APOE* ε4 carrier status, carriers showed faster progression for all biomarkers (Figure 6).

## Discussion

In the present study, we developed a model that allows for the mapping of the temporal trajectories of cortical Aβ, striatal Aβ, and tau protein deposition according to sex and *APOE* genotype from a large sample of ADNI subjects. Our major findings are as follows: First, Aβ was found to accumulate in the cortex and then in the striatum, followed by pathological tau accumulation in the limbic regions, which correspond to the Braak stages III and IV. Second, while there was no difference in cortical Aβ accumulation rate between women and men, striatal Aβ accumulation was found to be faster in women than in men, and this temporal difference according to sex was more pronounced in tau accumulation. Finally, *APOE* ε4 carriers showed faster accumulation of cortical Aβ, striatal Aβ, and tau than non-carriers. Taken together, our findings suggest that our temporal trajectory models can reveal a distinctive progression pattern of AD biomarkers depending on sex and *APOE* genotype. Therefore, our trajectory models can contribute to the design of personalized treatment and prevention strategies for AD in clinical practice.

Our finding that Aβ accumulates in the cortex and striatum sequentially is in line with Thal Aβ pathology staging, with Aβ deposits found initially in the cortex, and subsequently in the striatum (Thal et al., 2002), as also shown in a previous imaging study (Cho et al., 2018). Our other finding regarding the order of striatal Aβ and tau accumulation is also consistent with previous pathological studies showing that striatal Aβ plaques predicted the possible development of higher Braak stages (Braak and Braak, 1990; Beach et al., 2012). Therefore, our findings are consistent with the Aβ pathological cascade hypothesis. Regarding cortical Aβ, the time to reach the abnormal SUVR cutoff was 14.9 years in the present study, but 6.4 years in the previous study (Jagust et al., 2021). This discrepancy might be explained by the differences in the subjects. In the previous study, subjects with Aβ-negative normal cognition at baseline with increasing ^18^F-florbetapir slopes were used as a control group (Jagust et al., 2021). By contrast, in the present study, subjects who were consistently Aβ-negative cognitively normal during the follow-up period were included as a control group. However, the time to reach the abnormal cortical Aβ SUVR cutoff of the present study (14.9 years) is closer to that of previous studies (12-16 years) (Villemagne et al., 2013; Baek et al., 2020a). For the tau trajectory curve, the Baek et al. (2020a) estimate of the time interval to reach the *z*-score 2.0 for tau (30.6 years) was similar to our findings (22.7 years for the cutoff, *z*-score −2.0). Since striatal Aβ plaques are regarded as a predictor of higher Braak stages (Braak and Braak, 1990; Beach et al., 2012) and worse cognitive impairment (Beach et al., 2016; Grothe et al., 2017; Cho et al., 2018), it is important to take the striatal Aβ trajectory into account during clinical practice. Notably, our trajectory curve for striatal Aβ also calculated that it took 18.2 years to reach the abnormal SUVR cutoff. Thus, our study elucidating the temporal order in the accumulation of cortical Aβ, striatal Aβ, and pathological tau protein, along with their estimated time intervals, may support Thal Aβ pathology staging and the Aβ pathological cascade (Jack et al., 2010).

Several previous studies, including our group, showed that women had faster cognitive decline than men (Buckley et al., 2018; Cho et al., 2021), but that there was no such difference in terms of cortical Aβ accumulation, cross-sectionally (Hanseeuw et al., 2019) or longitudinally (Buckley et al., 2018). However, we found that this was different for striatal Aβ and tau accumulation. In particular, it was found that women took 2.7 years faster than men to reach the abnormal cut-off value of striatal Aβ trajectory. For tau accumulation, a previous longitudinal tau-PET study showed that tau protein accumulation is faster in women (Smith et al., 2020). We also found this to be the case in our study, where the temporal difference between the sexes was more pronounced in the tau trajectory than in the striatal Aβ trajectory. Specifically, tau accumulation was found to be 16.0 years faster in women than in men to reach the abnormal tau cut-off. Thus, our trajectory model showed that the rate of accumulation between sexes was different for each AD biomarker. Therefore, this faster accumulation of striatal Aβ and tau in women may explain the worse cognitive decline and greater frequency and prevalence of AD dementia in women.

Our final major finding was that, unlike the differences between sexes, the differences between *APOE* ε4 carriers and non-carriers showed a different pattern. In particular, for the *APOE* ε4 carriers, there was a faster decline in all three AD biomarker trajectories. Our findings are supported by previous studies showing that *APOE* ε4 carriers accumulate Aβ faster than non-carriers (Liu et al., 2013), as shown in amyloid PET (Ossenkoppele et al., 2015) and CSF studies (Resnick et al., 2015). Also, *APOE* ε4 carriers accumulated tau faster than non-carriers, as previously described (Sepulcre et al., 2018; Baek et al., 2020b). Since there were differences in AD biomarker trajectories depending on sex and *APOE* genotype, our findings will help to design individualized therapeutic and preventive strategies to ameliorate AD biomarkers, resulting in cognitive decline.

The ADNI is a well-organized, longitudinal cohort that serves as an excellent resource for investigating the disease course of AD with multimodal imaging markers, including Aβ and tau PET. However, this study had several limitations. First, the number of subjects who underwent tau PET was small compared to the number of subjects who underwent amyloid PET. Second, we defined the time 0 for a specific biomarker as the point when the SUVR value of that biomarker was in the mean level of Aβ-negative CN subjects. We were able to estimate the durations for each biomarker to reach certain values, but we need to synchronize the time axes between biomarkers in future studies. Also, further studies with long-term longitudinal follow-up are needed to validate our findings. Since statistical comparison was difficult, we explained the time comparison of each graph descriptively, as in other trajectories studies (Baek et al., 2020a; Jagust et al., 2021). Third, we did not investigate the interactive effects of gender and *APOE* genotype on the biomarkers’ trajectories because of a small sized sample. Further studies with a larger sized sample are needed to evaluate this issue. Nevertheless, our study is noteworthy in that we demonstrated that trajectory curves of AD biomarkers differ according to striatal Aβ involvement, sex, and *APOE* genotype.

In conclusion, the temporal trajectory in this study reflects the Thal Aβ pathology staging and the amyloid cascade hypothesis, showing that pathological tau protein accumulation occurred only when striatal Aβ accumulation emerged. Furthermore, trajectory curves differed according to sex and *APOE* genotype. In particular, a similar or higher rate of cortical Aβ accumulation in men compared to women was reversed with respect to striatal Aβ and tau accumulation. In clinical practice, the prognoses for AD patients should be approached differently in relation to striatal Aβ involvement, sex, and *APOE* genotype.

## Data Availability Statement

The original contributions presented in the study are included in the article/supplementary material, further inquiries can be directed to the corresponding author/s. The datasets generated during the study are available in the http://adni.loni.usc.edu/data-samples/access-data/.

## Ethics Statement

The Ethics committees/institutional review boards that approved the ADNI study are: Albany Medical Center Committee on Research Involving Human Subjects Institutional Review Board, Boston University Medical Campus and Boston Medical Center Institutional Review Board, Butler Hospital Institutional Review Board, Cleveland Clinic Institutional Review Board, Columbia University Medical Center Institutional Review Board, Duke University Health System Institutional Review Board, Emory Institutional Review Board, Georgetown University Institutional Review Board, Health Sciences Institutional Review Board, Houston Methodist Institutional Review Board, Howard University Office of Regulatory Research Compliance, Icahn School of Medicine at Mount Sinai Program for the Protection of Human Subjects, Indiana University Institutional Review Board, Institutional Review Board of Baylor College of Medicine, Jewish General Hospital Research Ethics Board, Johns Hopkins Medicine Institutional Review Board, Lifespan - Rhode Island Hospital Institutional Review Board, Mayo Clinic Institutional Review Board, Mount Sinai Medical Center Institutional Review Board, Nathan Kline Institute for Psychiatric Research & Rockland Psychiatric Center Institutional Review Board, New York University Langone Medical Center School of Medicine Institutional Review Board, Northwestern University Institutional Review Board, Oregon Health and Science University Institutional Review Board, Partners Human Research Committee Research Ethics, Board Sunnybrook Health Sciences Centre, Roper St. Francis Healthcare Institutional Review Board, Rush University Medical Center Institutional Review Board, St. Joseph’s Phoenix Institutional Review Board, Stanford Institutional Review Board, The Ohio State University Institutional Review Board, University Hospitals Cleveland Medical Center Institutional Review Board, University of Alabama Office of the IRB, University of British Columbia Research Ethics Board, University of California Davis Institutional Review Board Administration, University of California Los Angeles Office of the Human Research Protection Program, University of California San Diego Human Research Protections Program, University of California San Francisco Human Research Protection Program, University of Iowa Institutional Review Board, University of Kansas Medical Center Human Subjects Committee, University of Kentucky Medical Institutional Review Board, University of Michigan Medical School Institutional Review Board, University of Pennsylvania Institutional Review Board, University of Pittsburgh Institutional Review Board, University of Rochester Research Subjects Review Board, University of South Florida Institutional Review Board, University of Southern, California Institutional Review Board, UT Southwestern Institution Review Board, VA Long Beach Healthcare System Institutional Review Board, Vanderbilt University Medical Center Institutional Review Board, Wake Forest School of Medicine Institutional Review Board, Washington University School of Medicine Institutional Review Board, Western Institutional Review Board, Western University Health Sciences Research Ethics Board, and Yale University Institutional Review Board. The patients/participants provided their written informed consent to participate in this study.

## Author Contributions

JK, MC, and SS: conceptualization and methodology. JK and SK: formal analysis and investigation. JK and MC: writing – original draft preparation. HJ, HK, JJ, and SS: writing – review and editing. SS: funding acquisition. DN and SS: supervision. All authors contributed to manuscript revision, read, and approved the submitted version.

## Conflict of Interest

The authors declare that the research was conducted in the absence of any commercial or financial relationships that could be construed as a potential conflict of interest.

## Publisher’s Note

All claims expressed in this article are solely those of the authors and do not necessarily represent those of their affiliated organizations, or those of the publisher, the editors and the reviewers. Any product that may be evaluated in this article, or claim that may be made by its manufacturer, is not guaranteed or endorsed by the publisher.

## Footnotes

1
http://www.adni-info.org
